# Preliminary Outcomes in Care Partners of Patients receiving Lecanemab

**DOI:** 10.1002/alz70859_105196

**Published:** 2025-12-25

**Authors:** Clarissa M Howe, Leah R Hanson, Marcel G Hungs, Samantha J Sherman, Lauryn E Jones, Luke R Conway, Bhavani Kashyap

**Affiliations:** ^1^ HealthPartners Center for Memory & Aging, St. Paul, MN USA; ^2^ HealthPartners Institute, Minneapolis, MN USA; ^3^ HealthPartners Center for Memory & Aging, St Paul, MN USA; ^4^ HealthPartners Institute, Bloomington, MN USA; ^5^ HealthPartners Center for Memory & Aging, Saint Paul, MN USA

## Abstract

**Background:**

Secondary analysis of the CLARITY‐AD (Lecanamab) trial suggests less increase in burden in Care Partners (CP) and less decline in quality of life in persons living with dementia (PLWD) (Cohen et al 2023). In this pilot study we are exploring CP outcomes as part of a registry for PLWD who receive lecanemab infusions at a multidisciplinary neurology clinic.

**Method:**

PLWD and their CP were enrolled prior to receiving Lecanamab infusions through clinic referrals. Enrolled CP complete self‐reported measures at multiple times [baseline (prior to start of infusion), 3 months (M), 6M, 12M, and 18M visits following start of infusions] and a semi‐structured interview at 3M visit to gather PLWD and CP experience. We present preliminary data from our on‐going study with baseline and 3M visits. Self‐reported measures included questions on sleep (Pittsburgh Sleep Quality Index (PSQI), caregiver burden (12 item ‐ Zarit Burden Interview (ZBI‐12) and experience.

**Results:**

Out of the 13 patients contacted, 10 were enrolled and 3 declined participation (logistics (n=2); time commitment (n=1)). Two patients were withdrawn (one, stopped infusions due to anxiety and other was lost to follow up). Among the 10 CP that completed baseline, 60% (n=6) were male, 100% were spouses of PLWD (n=10) and mean age was 70.6 years at enrollment. At baseline, mean CP global PSQI was 9.5 (SD:4.6) and burden was 15.4 (SD:9.7). Of the 6 that completed the 3M visit, we noted no increase in CP burden, while 50% of CP (n=3) reported less burden than at baseline. With regards to sleep, 67% of CP showed poor sleep efficiency based on global PSQI score at both visits, though scores showed a reduction at the 3M visit. All participants recommended infusions to others and 33% CP (n=2) perceived positive changes in terms of memory or daily activities or behavior in PLWD.

**Conclusion:**

Lecanemab infusions was well accepted in CP and PLWD with overall experience being positive following 3M infusions. Additionally, this did not add burden to CP. On‐going data collection will provide further insights into CP outcomes.

